# Intracellular Freezing in the Infective Juveniles of *Steinernema feltiae*: An Entomopathogenic Nematode

**DOI:** 10.1371/journal.pone.0094179

**Published:** 2014-04-25

**Authors:** Farman Ali, David A. Wharton

**Affiliations:** Department of Zoology, University of Otago, Dunedin, New Zealand; University of California at Berkeley, United States of America

## Abstract

Taking advantage of their optical transparency, we clearly observed the third stage infective juveniles (IJs) of *Steinernema feltiae* freezing under a cryo-stage microscope. The IJs froze when the water surrounding them froze at −2°C and below. However, they avoid inoculative freezing at −1°C, suggesting cryoprotective dehydration. Freezing was evident as a sudden darkening and cessation of IJs' movement. Freeze substitution and transmission electron microscopy confirmed that the IJs of *S*. *feltiae* freeze intracellularly. Ice crystals were found in every compartment of the body. IJs frozen at high sub-zero temperatures (−1 and −3°C) survived and had small ice crystals. Those frozen at −10°C had large ice crystals and did not survive. However, the pattern of ice formation was not well-controlled and individual nematodes frozen at −3°C had both small and large ice crystals. IJs frozen by plunging directly into liquid nitrogen had small ice crystals, but did not survive. This study thus presents the evidence that *S*. *feltiae* is only the second freeze tolerant animal, after the Antarctic nematode *Panagrolaimus davidi*, shown to withstand extensive intracellular freezing.

## Introduction

The entomopathogenic nematode *Steinernema feltiae* is a temperate species and is globally distributed [1]. In cold regions the infective free-living third-stage juveniles (IJs) of this nematode are exposed to varying and to sub-zero temperatures. Nematodes are aquatic [2] and are at risk of inoculative freezing by ice in their surroundings [3], penetrating through body openings such as the mouth or anus [4]. Some studies suggest that *S. feltiae* is a cold tolerant species [5]. However, the underlying mechanism of cold survival in entomopathogenic nematodes, including this species, is poorly understood.

In our previous experiments, we showed that *S. feltiae* IJs could survive sub-zero temperatures [6] but not whether the nematodes themselves or just the water surrounding them froze. Here we determine the mechanism of cold tolerance of *S. feltiae* IJs based on morphological changes at the cellular level after freezing them under various conditions. Two techniques have been used: cold stage microscopy and freeze substitution/transmission electron microscopy. Nematodes are transparent and freezing within their bodies can be observed under a cold microscope stage [5], [7], [8]. This allows us to observe whether or not the nematode freezes. Shrinkage of the nematode due to loss of water can also be observed. This occurs in situations where the water surrounding a nematode freezes but not that within its body; a process called ‘cryoprotective dehydration’ [9].

Freeze substitution is a process in which the ice within the frozen specimen is replaced by an organic solvent, such as methanol or acetone, at a very low temperature; below −70°C [10]. The technique is usually used for specimens that have been rapidly frozen and is followed by embedding in resin. Embedding permits cutting of ultrathin sections for electron microscopy. Rapid freezing of a biological specimen aims to prevent ice formation through water vitrification and thus provides better preservation of ultrastructure, and the retention of water-soluble components and antigenicity, than is achievable using more conventional chemical fixation [11]. However, the technique can also be used to visualize the position of ice formed in samples frozen more slowly [12], [13], [14]. We used methanol as a substitution solvent because this has a high capacity for water absorption, can dissolve sugar and is liquid at a substitution temperature (−90°C) which does not allow ice recrystallization [15]. Fixatives dissolved in the substitution fluid infiltrate into the sample and react with its proteins, lipids and other constituents when the temperature is raised sufficiently for rapid cross-linking to occur. Embedding in resin is then carried out, replacing the substitution fluid. The previous position of ice is visible as a white space. Freeze substitution provides accurate information about the distribution of ice in both extra and intracellular compartments.

We used freeze substitution of nematodes after different freezing regimes involving different temperatures and exposure times. The aim was not to prevent ice formation but to locate the position and pattern of ice crystals within the frozen nematodes, which may account for differences in their freezing survival.

## Materials and Methods

### Nematode culture

*Steinernema feltiae* was grown in *Galleria mellonella* larvae at 23°C using the method described by Kaya and Stock [16]. The fresh IJs were used for experiments on the same day they were collected.

### Cold stage microscopy

The freezing process was observed on a microscope cold stage similar to that described by Wharton & Rowland [8]. This is based on a thermoelectric cooling (Peltier) module, the hot face of which is cooled by circulating fluid from a Haake F3-Q refrigerated circulator (Thermo Fisher Scientific, Waltham, MA) and which is mounted on a Zeiss Axiophot Photomicroscope (Carl Zeiss Inc., Thornwood, NY). Nematode samples in a 1 μl drop of artificial tap water (ATW) [17] were mounted between two small glass coverslips in the sample chamber of the cold stage. The temperature was monitored with a NiCr/NiAl thermocouple inserted in the metal finger of the cold stage close to the specimen via a Comark electronic thermometer. The electronic thermometer was calibrated against the melting point of deionized water. Samples were cooled from 2°C to various minimum temperatures (−1, −2, −3, −4, −5, −6°C) at 0.5°C min^−1^ and freezing was initiated by seeding with a small ice crystal at the edge of the coverslip. Nitrogen gas was supplied to the sample chamber of the cold stage to prevent condensation. The freezing process was visualized using a 10× objective lens and images were captured at regular intervals by a Canon Powershot A640 Digital Camera, controlled by Axio Vision v. 4.6 software (Zeiss) run on an Insite PC. Freezing of nematodes was observed as a sudden darkening. Unfrozen nematodes did not change their appearance and remained clear. After seeding, the sample was allowed to stay frozen for either 3 h at −1°C or 30 min at all other test temperatures, and then rewarmed to 2°C at 0.5°C min^−1^. Nematodes were removed from the cold stage once it had reached room temperature and transferred to watch glasses in ATW. Survival was determined after 24 hours. The experiment was replicated three times.

### Freezing regimes used for freeze substitution

To see the differences in morphology of IJs after freezing, different freezing regimes were used. Each sample consisted of 10 μl of nematode suspension in an Eppendorf tube (four replicates, two separate cultures) transferred to a cooling block, the temperature of which was controlled by fluid circulating from a Haake Phoenix II-C35P programmable refrigerated circulator (Thermo Fisher Scientific). No thermocouple was inserted into samples processed for freeze substitution. Fast-frozen samples were cooled from 2°C to −3°C or −10°C at 0.5°C min^−1^, seeded with an ice crystal and held at −3°C or −10°C for 75 min. Slow-frozen samples were cooled from 2°C to −1°C at 0.5°C min^−1^, seeded with an ice crystal and held at −1°C for either 75 min or overnight. At the end of the freezing time, it was checked that the samples were frozen and they were then transferred to liquid nitrogen for transportation before freeze substitution.

Freezing survival was determined from samples subjected to the same freezing regimes as above, but with a NiCr/NiAl thermocouple inserted in each tube. Thermocouples were interfaced to a Macintosh computer via a Powerlab A/D interface (ADInstruments Ltd., Dunedin, NZ). The temperature records were analysed using a computer programme (Chart v3.2.7, ADInstruments) to ensure that the exotherm, and hence the freezing of the sample was completed. The duration of the exotherm was used as an indication of its freezing rate. Samples were rewarmed at 0.5°C min^−1^ to 2°C and transferred to ATW in watchglasses at room temperature. Survival was determined after 24 hours by counting the proportion moving after a mechanical stimulus.

Nematode suspensions in Eppendorf tubes were also plunged directly into liquid nitrogen without prior freezing and processed via freeze substitution to serve as a frozen control. Nematodes were also prepared for electron microscopy using a conventional preparation technique to compare the normal nematode morphology with those that were frozen. This consisted of fixing them in 4% formaldehyde, 2.5% glutaraldehyde, 1% osmium tetroxide followed by dehydration in ethanol, and embedding in Spurr resin.

### Freeze substitution and embedding

Samples in liquid nitrogen were transferred into a Reichert automatic freeze substitution (AFS) apparatus and substitution fluid (methanol containing fixatives) added. Freeze substitution and embedding were then carried out as reported previously [14]. Ultrathin (80 nm) sections were cut with a Reichert ultramicrotome, stained with uranyl acetate and lead citrate and observed using a Philips CM100 Transmission Electron Microscope, operated at 80 kV. The freeze substitution experiment was repeated three times.

### Analysis of ice crystals

The following parameters were measured after transmission electron microscopy.

1. Areal Fractions (A_a_) were calculated using a stereological method based on point counting [18]. An A4 size transparent sheet containing a grid of crosses (+) was superimposed on the entire transverse section image. Areal fraction was taken to be the ratio of the crosses hitting the ice crystals to the crosses hitting the section. This was determined by the formula A_a_ = ∑P_c_/∑P_s_ (where A_a_ =  areal fraction, ∑P_c_ =  total points hitting the ice crystals and ∑P_s_ =  total points hitting the section).
2. Ice crystal diameter of the five largest ice crystals in each section of all the treatments was measured using ImageJ software [19].
3. Ice crystal pattern: frozen nematodes having all small, all large or a mixture of both were counted.
4. Shrinkage due to partial dehydration was compared between freezing regimes. This was visible in sections where the regular elliptical or circular profile was lost.

Nematodes were also examined for the presence of intracellular ice (ice surrounded by cytoplasm), extracellular ice (ice confined to the pseudocoel, intestinal lumen, and/or extracellular spaces), and those that were unfrozen. All images taken (13–46 per treatment) were used for quantifying ice crystal pattern and shrinkage of nematodes (parameters 3, 4). However, measurements of the diameter of the five largest ice crystals and areal fractions (parameters 1, 2) were confined to those sections which were well infiltrated with resin and were clear enough to measure these parameters accurately (13–24 per treatment).

### Statistical analysis

The Statistical Package for Social Sciences (SPSS) ver. 15.0 was used for statistical analyses [20]. Areal fractions were arcsine transformed before analysis. Measurements of the five largest crystals from each section were averaged before analysis to give a single measurement. A one way analysis of variance (ANOVA) was conducted to see if the differences among the freezing regimes for various parameters were significant. Tukey's test was used to compare means. Where variances were not equal after running Leven's test of homogeneity of variances the Brown-Forsythe and Welch tests were used instead of ANOVA. Means were then separated using the Games-Howell post-hoc multiple comparison test.

## Results

### Cold stage observations

**Figure 1:**
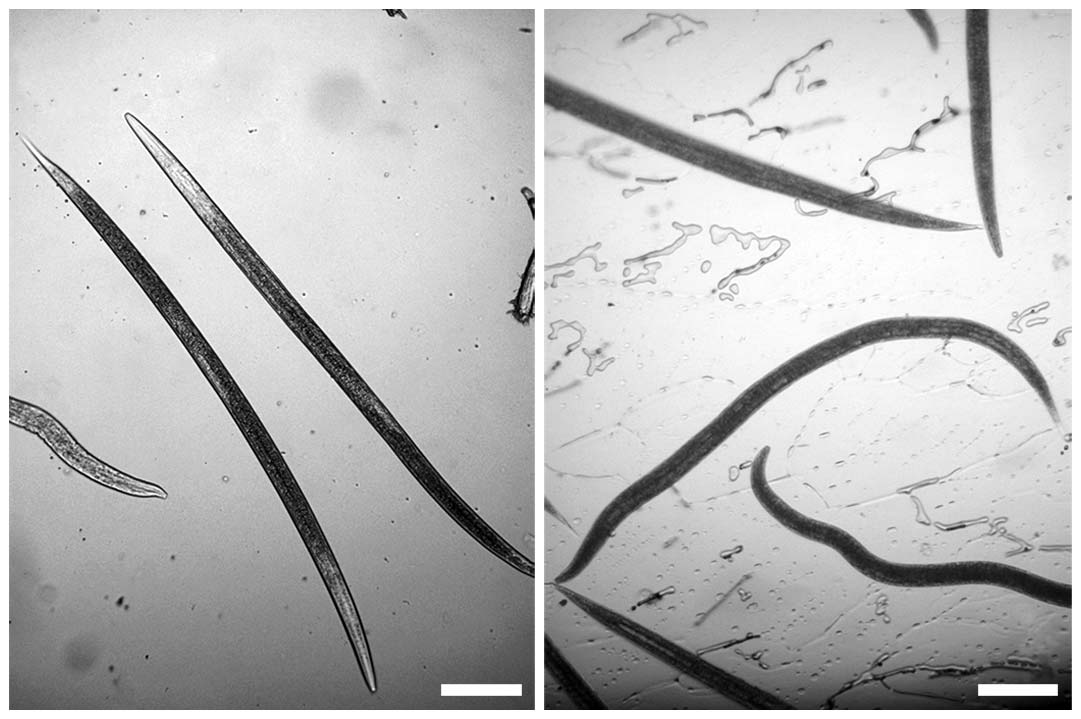
Infective juveniles of *Steinernema feltiae*: transparent nematodes are unfrozen 3 hours after seeding the ice crystal at −1°C (left); darkened nematodes are frozen 30 min after seeding at −2°C and below (right). Scale bars  =  100 μm.

**Figure 2:**
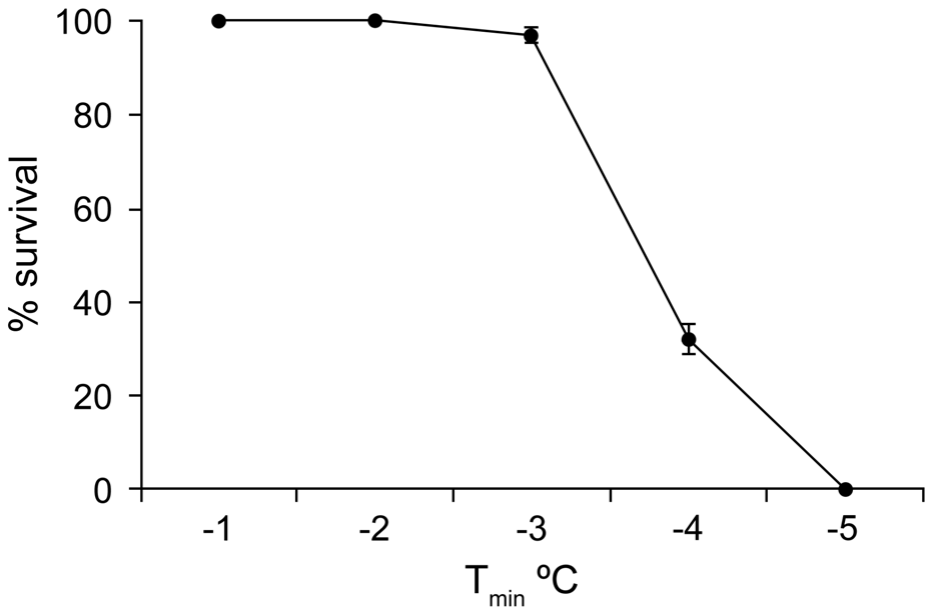
Survival of the infective juveniles of *S*. *feltiae* at various minimum temperatures (T_min_) after freezing them on the microscope cold stage. Vertical bars represent the standard errors of the means of 3 measurements.

At −2°C and below, nematodes were clearly observed to freeze by exogenous ice nucleation by the surrounding ice, as indicated by a sudden darkening of the nematodes. Exogenous ice nucleation did not occur at −1°C and nematodes remained clear and unfrozen, although the medium surrounding them was frozen (Fig 1). Nematodes survived the freezing process down to −4°C. Nematode survival was better at high sub-zero temperatures (−1, −2, −3°C) than at −4°C (Fig. 2). There was no difference in survival between frozen and unfrozen nematodes at temperatures −1 to −3°C. Also there was no difference in the appearance of frozen nematodes between the test temperatures (−2 to −5°C).

### Freeze substitution experiments

**Figure 3:**
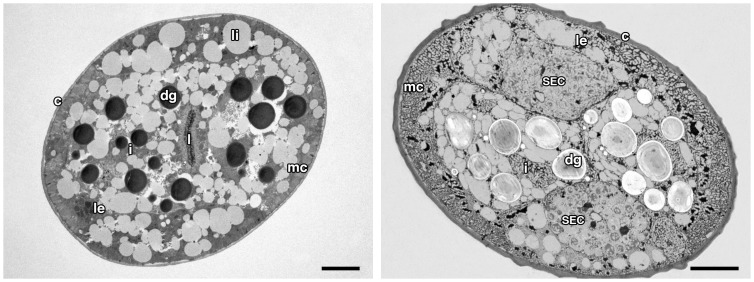
Transmission electron micrograph of transverse sections of the IJ of *Steinernema feltiae* prepared through conventional chemical fixation (left) and by freeze substitution after freezing in liquid nitrogen, note the small ice crystals throughout the tissues (right). c, cuticle; mc, muscle cell; dg, dense granules; le, lateral epidermal cord; i, intestinal cell; l, intestinal lumen; li, lipid droplet; SEC, secretary-excretory cell. Scale bars  =  5 μm.

The morphology of an unfrozen *S*. *feltiae* IJ sectioned through the intestinal region after preparation for transmission electron microscopy by chemical fixation is shown in Fig. 3. The outermost layer is the cuticle, underneath which is a thin epidermis and then a layer of longitudinal muscles. Out of the four epidermal cords (two lateral, mid dorsal and mid ventral), the most prominent are the lateral epidermal cords. The intestinal lumen and the microvilli are clearly visible in the middle region. The dark circular structures are dense food storage granules. The cytoplasmic region is characterized by the presence of mitochondria. The pseudocoel is very thin and is not distinguishable in the image.

The morphology of specimens frozen in liquid nitrogen and prepared by freeze substitution for electron microscopy is similar to that of non-frozen controls except for its reticulated appearance due to the presence of small ice crystals throughout the tissues, including cell organelles (Fig. 3). The circular structures are dense food storage granules similar to those seen in unfrozen controls but they have a paler appearance after freezing and freeze substitution. A similar appearance observed in samples from other temperature treatments to that of those frozen directly in liquid nitrogen was taken to indicate that nematodes were unfrozen before immersion in liquid nitrogen.

**Figure 4:**
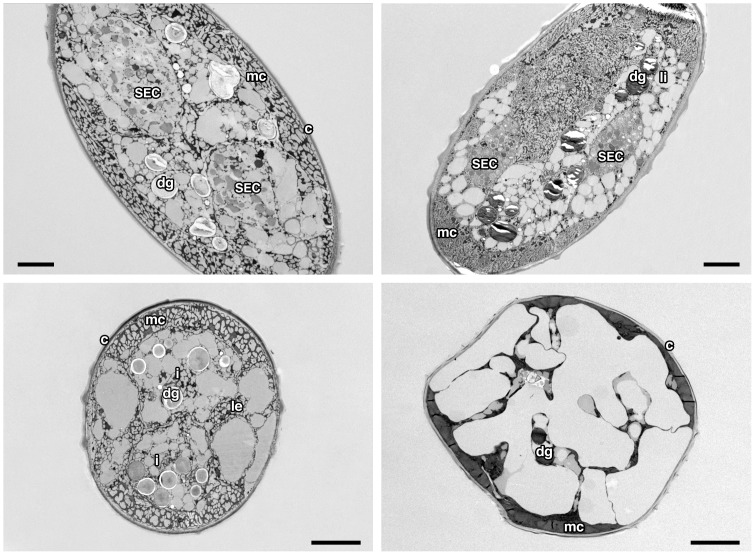
Transmission electron micrograph of a transverse section of the third stage IJ of *S*. *feltiae* frozen at −1°C for 75 min (top left), −1°C overnight (top right), −3°C for 75 min (bottom left) or −10°C for 75 min (bottom right); and prepared by freeze substitution. c, cuticle; mc, muscle cell; dg, dense granules; i, intestinal cell; le, lateral epidermal cord; li, lipid droplet; SEC, secretary-excretory cell. Scale bars  =  5 μm.

**Figure 5:**
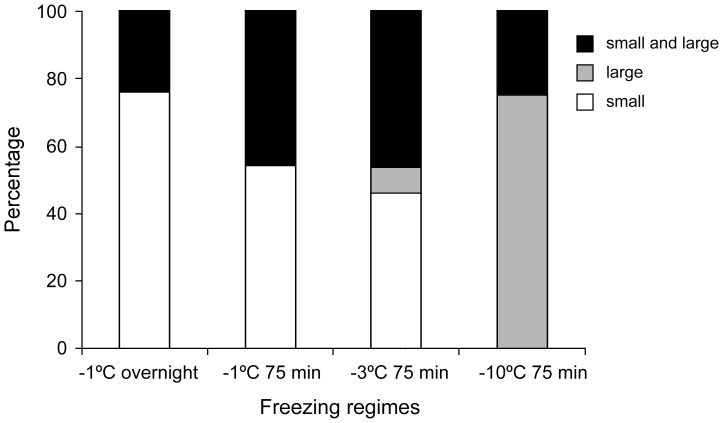
The percentage of IJs of *S*. *feltiae* having all small, all large and a mixture of small and large ice crystals in transverse sections.

Nematode survival was high after freezing at −1°C or −3°C for 75 min and −1°C overnight but there was no survival after freezing at −10°C (Table 1). Three different ice crystal patterns were observed in nematodes after subjecting them to slow and fast freezing. Specimens with all small, all large or a mixture of small and large ice crystals were found (Fig. 4). Small ice crystals were found in nematodes frozen at −1°C. Ice crystals were larger in those frozen at −3°C and larger still in those frozen at −10°C (Fig. 4). The percentage of nematodes showing all small, all large or a mixture of ice crystals is shown in Fig. 5. Ice crystals were observed in both intracellular and extracellular compartments in all the freezing regimes and were prominent in the intestinal cells, pharynx, secretory-excretory cells and muscle cells. Sections with small ice crystals appeared slightly shrunken compared to those with large ice crystals.

**Table 1:** Exotherm durations and survival of *S*. *feltiae* samples (*N* = 4).

Treatment	Exotherm duration (min)	Survival (%)
−1°C overnight	65.1±1.2	100
−1°C for 75 min	64.6±0.1	100
−3°C for 75 min	24.6±0.4	97.5±0.9
−10°C for 75 min	4.5±0.8	0
Liquid nitrogen	—	0
Non-frozen control	—	100

means ± SE.

### Measurements on transverse sections

**Figure 6:**
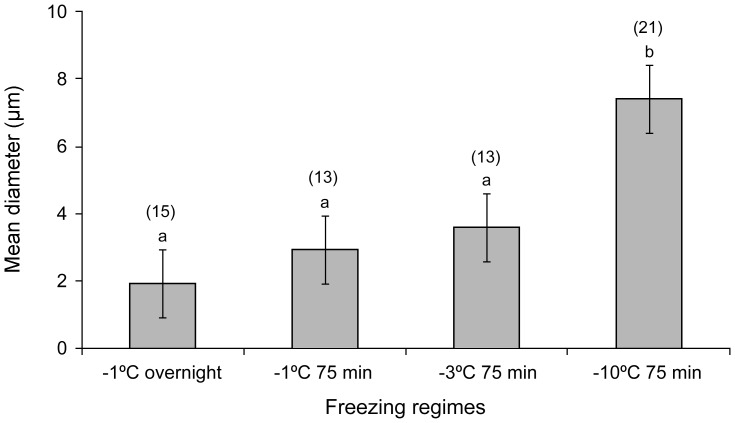
Diameters of the five largest ice crystals of transverse sections frozen overnight at −1°C, or for 75 min frozen at −1°C, −3°C, and −10°C. Vertical bars represent standard errors of means. The number of sections measured in each treatment is shown in parenthesis. Different small letters above each bar indicate that the treatments are significantly different (P<0.05).

**Figure 7:**
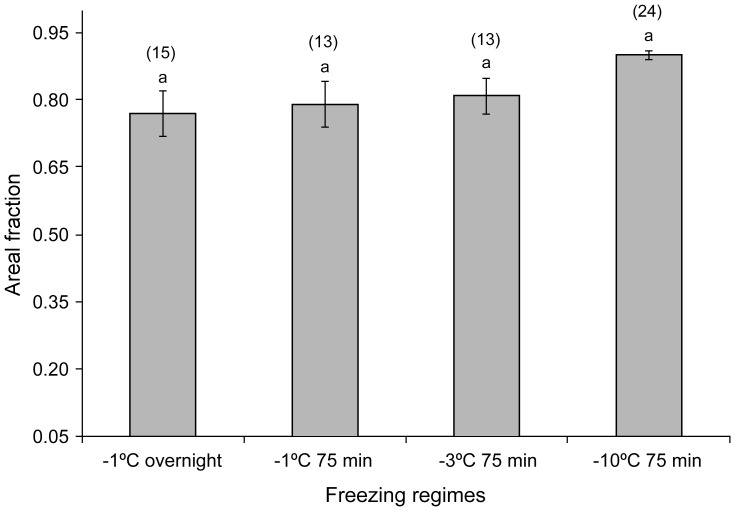
Changes in areal fraction of ice crystals in transverse sections frozen overnight at −1°C, or for 75 min frozen at −1°C, −3°C, and −10°C. Vertical bars represent standard errors of means. The number of sections measured in each treatment is shown in parenthesis. Different small letters above each bar indicate that the treatments are significantly different (P<0.05).

**Figure 8:**
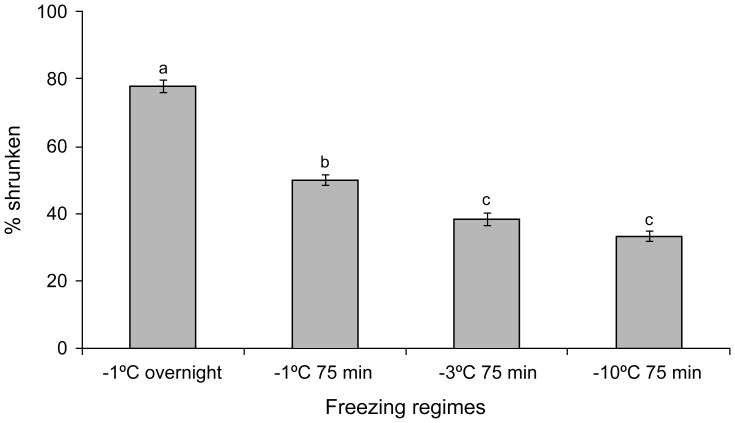
Percentage of the nematodes slightly shrunken after freezing at −1°C overnight or for 75 min frozen at −1°C, −3°C and −10°C. Vertical bars represent standard errors of means (N = 3). Different small letters above each bar indicate that the treatments are significantly different (P<0.05).

Overall there was a significant difference between the diameters of the five largest ice crystals measured between freezing regimes (Fig. 6: F_3, 58_ = 16.45; P<0.05). Post hoc comparison tests showed that ice crystal diameters in nematodes frozen at −10°C were significantly different from those frozen at −1°C and −3°C. However, ice crystal diameters did not differ significantly between nematodes frozen at −1°C overnight, −1°C and −3°C for 75 min (Fig. 6). Analysis of variance showed that areal fractions of the ice crystals did not vary significantly (F_3, 60_ = 2.1; P>0.05) among the freezing regimes (Fig. 7). No severe shrinkage of the tissues was noticed, since all the nematodes had ice crystals in their tissues. However, slight shrinkage of nematode tissues due to partial dehydration varied significantly among the treatments (F_3, 12_ = 135.7; P<0.05) and was more prominent in the slow freezing regimes (−1°C: 50–77.8%) than the fast freezing regimes (−3°C, −10°C: 33.3–38.5%) as shown in Fig. 8.

## Discussion

Nematodes are transparent and thus the freezing process was clearly observed on the microscope cold stage. At −2°C and below the infective juveniles of *S*. *feltiae* froze by inoculative freezing from the external medium. None of the infective juveniles were frozen before the surrounding water froze. Inoculative freezing in nematodes is not uncommon, since they are always in contact with water and has been reported in several studies [7], [21], [5]. The sudden darkening coupled with immobilization of nematodes was an indication of freezing. The darkening may be due to the scattering of light by the ice. Darkening upon freezing in nematodes has been reported in several nematodes species such as *Aphelenchoides ritzemabosi* [22], *Meloidogyne* spp. [23], *Wetanema* sp. [21], *Steinernema* spp. and *Heterorhabditis* spp. [5], *Anisakis* sp. [24], *Panagrolaimus davidi* [9] and *Caenorhabditis elegans* [25].

Inoculative freezing did not occur at −1°C in *S. feltiae* and nematodes did not freeze at this temperature, although the water surrounding them was frozen. There was no change in appearance or any sign of darkening after seeding with an ice crystal, indicating that they did not freeze (Fig. 1). This indicates some low level of resistance by this species against inoculative freezing at high sub-zero temperature. The mechanism of this resistance at −1°C is not clear but it would facilitate cryoprotective dehydration, where the nematodes remain unfrozen and lose water to the surrounding ice [9]. Cryoprotectants such as glycerol and trehalose are accumulated by entomopathogenic nematodes before freezing [26] and could protect the nematodes from freezing at high sub-zero temperature (−1°C), since they raise the osmolality and thus lower the melting point of the body fluids. *S*. *feltiae* may have the ability to supercool its body fluids and be freeze avoiding at high sub-zero temperature (−1°C), similar to the cold tolerance strategy of *Heterorhabditis zealandica* [27].

At −2°C, and lower temperatures, all the nematodes turned dark and stopped moving, suggesting that their survival in the cooling block experiments at these temperatures [6] was due to freezing tolerance. Cold stage observations on *S. feltiae* have been reported by Brown and Gaugler [5]. Our findings are in partial agreement with their results. They have reported that the infective juveniles of entomopathogenic nematodes, including *S*. *feltiae*, were freeze tolerant and froze as the temperature decreased after initiating freezing at −0.5°C. However, it is not clear from their study at which temperature the nematode froze, and they did not provide any pictures of the freezing event, so their nematodes may not have frozen at −1°C. The results of the present study are similar to those reported by Wharton et al. [9] from the Antarctic nematode *Panagrolaimus davidi*, which does not freeze if the medium is seeded at −1°C and undergoes cryoprotective dehydration, but freezes below −2°C. A small amount of shrinkage, that may indicate cryoprotective dehydration, was observed in *S. feltiae* frozen at −1°C. Some nematode species freeze even at −1°C and cannot avoid inoculative freezing [28].

Freeze substitution enabled the location of ice crystals to be determined, appearing as white spaces after the frozen water was replaced by substitution fluid and then by resin inside the frozen nematodes. Overall, fast-frozen samples had large and slow-frozen samples had small ice crystals. Ice crystals were observed both in intracellular and extracellular compartments. Nematodes frozen directly in liquid nitrogen had small ice crystals but did not survive (Fig. 4). They had a reticulated appearance indicating the formation of small ice crystals rather than an amorphous (vitrified or glassy) state [11]. None of the freezing regimes used in this study would be fast enough to produce vitrification. Water needs to be cooled at a rate greater than 10,000°C s^−1^ for vitrification to occur [29]. Nematodes frozen at −1°C for either 75 min or overnight had very small ice crystals similar to those frozen in liquid nitrogen. Nematodes do not freeze at −1°C (as observed earlier on the microscope cold stage) and their water would freeze rapidly when plunged into liquid nitrogen, producing the observed pattern of small ice crystals.

Slight shrinkage is evident in some treatments. This indicates partial dehydration and was noticeably greater in the samples frozen overnight at −1°C than the rest of the treatments. This is an indication of cryoprotective dehydration as water is lost from liquid water inside the nematode to the surrounding ice, as a result of the difference in vapour pressure [30]. However, the evidence for cryoprotective dehydration in *S. feltiae* is not as strong as that described in an Antarctic nematode *P*. *davidi* [14] where no crystals were formed in slow-frozen samples and the nematodes appeared severely shrunken. Unfrozen nematodes surrounded by ice also have a shrunken appearance in a free living nematode, *Panagrellus redivivus* [28].

Some organisms, such as the freeze tolerant wood frog *Rana sylvatica*, produce cryoprotectants during the freezing process itself as soon as ice nucleation is initiated which helps the organism survive sub-zero temperatures [31]. This also could explain why slow freezing of the infective juveniles of *S*. *feltiae* for an extended time (overnight) at −1°C may improve survival by allowing them to synthesize cryoprotectants.

Nematodes frozen at −3°C and −10°C were observed to have ice crystals in both intracellular and extracellular compartments and a different appearance from samples frozen in liquid nitrogen. Nematodes survived freezing at −3°C but not at −10°C. Ice crystals were observed in most of the compartments of the infective juveniles, including the pharynx, muscle cells, intestinal cells, secretary-excretory cells and epidermis. The pseudocoel was not clearly visible.

After thawing the infective juveniles were motile, pathogenic to their insect host as before and produced the next generation [6]. This suggests that *S*. *feltiae* is one of the rare organisms that have been shown to survive intracellular freezing. Most freezing tolerant animals can survive only extracellular ice formation and for them intracellular freezing is lethal [32]. The Antarctic nematode, *P*. *davidi* is the only organism previously reported to survive extensive intracellular freezing [4]. During freezing 82% of its body water is converted into ice, a much higher ice content than can be frozen in most freeze tolerant animals [33]. Some fat body cells in insects have also been observed to survive intracellular freezing but survival does not occur throughout the tissues of the insect [34], [35], [36]. The cell membrane is thought to be a barrier to ice nucleation in

mammalian cells [37], and intracellular ice formation is considered lethal [38]. However, Toner et al. [39] report that intracellular ice formation is catalysed by the plasma membrane itself by acting as a nucleating agent in the presence of external ice. Nematodes being essentially aquatic [2] are

frozen predominantly by heterogeneous ice nucleation indicating this nematode may have a

specialized cell membrane. Survival of intracellular freezing may depend upon the pattern of ice formation. The size of ice crystals in samples frozen at −3°C was significantly smaller than those frozen at −10°C which may account for their survival. More than 97% of nematodes survived at −3°C, whereas all the nematodes died at −10°C where all or the majority of ice crystals formed were large.

The formation of small ice crystals at −3°C could be due to the presence of an ice active protein, such as a recrystallization-inhibiting protein that inhibits the growth of the crystals [40]. Recrystallization is the growth of larger ice crystals at the expense of smaller ones, which if it occurs in a frozen organism could be lethal. Recrystallization inhibition (RI) may assist the survival of intracellular freezing in *S. feltiae*. Recrystallization inhibition has been reported in another species of entomopathogenic nematode *S*. *carpocapsae*, which was second in RI activity only to *P*. *davidi* when compared to four other species [41].

Although ice crystal size varied across the treatments, areal fractions were not significantly different, indicating no effect on the proportion of the body frozen. Fewer but larger ice crystals appear to be lethal to the nematodes irrespective of the proportional area they occupy. The formation of small ice crystals allows *S. feltiae* infective juveniles to survive freezing.

In conclusion, infective juveniles of *S*. *feltiae* resist inoculative freezing at high sub-zero temperature (−1°C). At lower sub-zero temperature intracellular freezing occurs, which the nematodes can survive, but only down to about − 4°C. The species however, cannot completely control their ice crystal formation, since some large crystals, as well as small crystals, are formed even at −3°C. Survival of intracellular freezing is associated with the smaller size of ice crystals formed during slow freezing, which may be controlled by a substance that inhibits recrystallization.

## References

[1] Hominick WM, Reid AP, Bohan DA, Briscoe BR (1996) Entomopathogenic nematodes: Biodiversity, geographical distribution and the convention on biological diversity. Biocontrol Science and Technology 6: 317–331.

[2] Wharton DA (1986) A functional biology of nematodes. London and Sydney: Croom Helm.

[3] Wharton DA (1995) Cold tolerance strategies in nematodes. Biological Reviews of the Cambridge Philosophical Society 70: 161–185. 21545388 10.1111/j.1469-185X.1995.tb01442.x

[4] Wharton DA, Ferns DJ (1995) Survival of intracellular freezing by the Antarctic nematode *Panagrolaimus davidi* . Journal of Experimental Biology 198: 1381–1387. 9319273 10.1242/jeb.198.6.1381

[5] Brown IM, Gaugler R (1996) Cold tolerance of Steinernematid and Heterorhabditid nematodes. Journal of Thermal Biology 21: 115–121.

[6] Ali F, Wharton DA (2013) Cold tolerance abilities of two entomopathogenic nematodes, *Steinernema feltiae* and *Heterorhabditis bacteriophora* . Cryobiology 66: 24–29. 23142823 10.1016/j.cryobiol.2012.10.004

[7] Wharton DA, Brown IM (1991) Cold tolerance mechanisms of the Antarctic nematode *Panagrolaimus davidi* . Journal of Experimental Biology 155: 629–641. 10.1242/jeb.00083 12477892

[8] Wharton DA, Rowland JJ (1984) A thermoelectric microscope stage for the measurement of the supercooling points of microscopic organisms. Journal of Microscopy-Oxford 134: 299–305.

[9] Wharton DA, Goodall G, Marshall CJ (2003) Freezing survival and cryoprotective dehydration as cold tolerance mechanisms in the Antarctic nematode *Panagrolaimus davidi* . Journal of Experimental Biology 206: 215–221. 12477892 10.1242/jeb.00083

[10] Shiurba R (2001) Freeze-substitution: Origins and applications. In: Shiurba R, Kwang WJ, editors. International review of cytology - A survey of cell biology. San Diego: Academic Press Inc. pp. 45–96. 10.1016/s0074-7696(01)06019-3 11407763

[11] Robards AW, Sleytr UB (1985) Low temperature methods in biological electron microscopy. Amsterdam: Elsevier Science. 572 p.

[12] Hunt CJ (1984) Studies on cellular structure and ice location in frozen organs and tissues: the use of freeze-substitution and related techniques. Cryobiology 21: 385–402. 6380949 10.1016/0011-2240(84)90077-4

[13] Raymond Ml, Wharton D (2013) The ability of the Antarctic nematode *Panagrolaimus davidi* to survive intracellular freezing is dependent upon nutritional status. Journal of Comparative Physiology B 183: 181–188. 10.1007/s00360-012-0697-0 22836298

[14] Wharton DA, Downes MF, Goodall G, Marshall C (2005) Freezing and cryoprotective dehydration in an Antarctic nematode (*Panagrolaimus davidi*) visualised using a freeze substitution technique. Cryobiology 50: 21–28. 15710366 10.1016/j.cryobiol.2004.09.004

[15] Muhlfeld C (2009) High-pressure freezing, chemical fixation and freeze-substitution for immuno-electron microscopy. Methods in Molecular Biology 611: 87–101. 10.1007/978-1-60327-345-9_7 19960324

[16] Kaya HK, Stock SP (1997) Techniques in insect nematology. In: Lacey LA, editor. Manual of techniques in insect pathology. San Diego, CA: Academic Press. pp. 281–324.

[17] Greenaway P (1970) Sodium regulation in freshwater mollusc *Limnaea stagnalis* (L) (Gastropoda, Pulmonata). Journal of Experimental Biology 53: 147–163. 5478671 10.1242/jeb.53.1.147

[18] Gundersen HJG, Bendtsen TF, Korbo L, Marcussen N, Moller A, et al (1988) Some new, simple and efficient stereological methods and their use in pathological research and diagnosis Acta Pathologica, Microbiologica et Immunologica Scandinavica. 96: 379–394. 10.1111/j.1699-0463.1988.tb05320.x 3288247

[19] Rasband WS (1997-2009) ImageJ. US national institutes of health, Bethesda, Maryland, USA. Available: httpt://rsb.info.nih.gov/ij.

[20] SPSS Inc (2006) SPSS for Windows, Release 15. SPSS Inc, Chicago, IL.

[21] Tyrrell C, Wharton DA, Ramløv H, Moller H (1994) Cold tolerance of an endoparasitic nematode within a freezing-tolerant orthopteran host. Parasitology 109: 367–372.

[22] Asahina E (1959) Frost-resistance in a nematode, *Aphelenchoides ritzemabos*i. Low Temperature Science, Series B 17: 51–62.

[23] Sayre RM (1964) Cold-Hardiness of nematodes. I. Effects of rapid freezing on the eggs and larvae of *Meloidogyne incognita* and *M*. *hapla* . Nematologica 10: 168–179.

[24] Wharton DA, Aalders O (2002) The response of *Anisakis* larvae to freezing. Journal of Helminthology 76: 363–368. 12498643 10.1079/JOH2002149

[25] Hayashi M, Oguma T, Amino H, Kita K, Murase N (2009) Freezing survival of the nematode *Caenorhabditis elegans* in the presence of a cryoprotectant. Cryobiology and Cryotechnology 15: 115–119.

[26] Jagdale GB, Grewal PS (2003) Acclimation of entomopathogenic nematodes to novel temperatures: trehalose accumulation and the acquisition of thermotolerance. International Journal for Parasitology 33: 145–152. 12633652 10.1016/s0020-7519(02)00257-6

[27] Wharton DA, Surrey MR (1994) Cold tolerance mechanisms of the infective larvae of the insect parasitic nematode *Heterorhabditis zealandica* Poinar. CryoLetters 15: 353–360.

[28] Hayashi M, Wharton DA (2011) The oatmeal nematode *Panagrellus redivivus* survives moderately low temperatures by freezing tolerance and cryoprotective dehydration. Journal of Comparative Physiology B 181: 335–342. 10.1007/s00360-010-0541-3 21153645

[29] Weimer RM (2006) Preservation of *C* *elegans* tissue via high-pressure freezing and freeze-substitution for ultrastructural analysis and immunocytochemistry. In: Strange K, editor. Methods in molecular biology *C elegans*: methods and application. Totowa, New Jersy: Humana Press Inc. pp. 203–221. 10.1385/1-59745-151-7:203 16988436

[30] Wharton DA (2011) Cold tolerance. In: Perry RN, Wharton DA, editors. Molecular and physiological basis of nematode survival. Wallingford: CABI Publishing. pp. 182–204.

[31] Storey JM, Storey KB (1985) Triggering of cryoprotectant synthesis by the initiation of ice nucleation in the freeze tolerant frog, *Rana sylvatica* . Journal of Comparative Physiology B 156: 191–195.

[32] Lee RE (1991) Principles of insect low temperature tolerance. In: Lee RE, Denlinger DL, editors. Insects at low temperature. New York/London: Chapman and Hall. pp. 17–46.

[33] Storey KB, Storey JM (1988) Freeze tolerance in animals. Physiological Reviews 68: 27–84. 3275942 10.1152/physrev.1988.68.1.27

[34] Lee RE, McGrath JJ, Todd Morason R, Taddeo RM (1993) Survival of intracellular freezing, lipid coalescence and osmotic fragility in fat body cells of the freeze-tolerant gall fly *Eurosta solidaginis* . Journal of Insect Physiology 39: 445–450.

[35] Salt RW (1962) Intracellular freezing in insects. Nature 193: 1207–1208.

[36] Salt RW (1959) Survival of frozen fat body cells in an insect. Nature 184: 1426–1426.14402719

[37] Grout BWW, Morris GJ (1987) Freezing and cellular organisation. In: Grout BWW, Morris GJ, editors. The Effects of Low Temperatures on Biological Systems. London: Edward Arnold. pp. 147–173.

[38] Muldrew K, Acker JP, Elliott JAW, McGann LE (2004) The water to ice transition: implications for living cells. Life in the Frozen State: 67–108.

[39] Toner M, Cravalho EG, Karel M (1990) Thermodynamics and kinetics of intracellular ice formation during freezing of biological cells. Journal of Applied Physics 67: 1582–1593.

[40] Wharton DA, Barrett J, Goodall G, Marshall CJ, Ramløv H (2005) Ice-active proteins from the Antarctic nematode *Panagrolaimus davidi* . Cryobiology 51: 198–207. 16102742 10.1016/j.cryobiol.2005.07.001

[41] Smith T, Wharton DA, Marshall CJ (2008) Cold tolerance of an Antarctic nematode that survives intracellular freezing: comparisons with other nematode species. Journal of Comparative Physiology B 178: 93–100. 10.1007/s00360-007-0202-3 17712562

